# Medical expenditures for fragility hip fracture in Japan: a study using the nationwide health insurance claims database

**DOI:** 10.1007/s11657-022-01096-8

**Published:** 2022-04-11

**Authors:** Takahiro Mori, Jun Komiyama, Tomoko Fujii, Masaru Sanuki, Keitaro Kume, Genta Kato, Yukiko Mori, Hiroaki Ueshima, Hiroki Matsui, Nanako Tamiya, Takehiro Sugiyama

**Affiliations:** 1 Health Services Research and Development Center, University of Tsukuba, 1-1-1 Tenno-dai, Tsukuba, Ibaraki 305-8575 Japan; 2 Department of General Medical Science, Graduate School of Medicine, Chiba University, Chiba, Chiba Japan; 3 Department of General Internal Medicine, Eastern Chiba Medical Center, Togane, Chiba Japan; 4 Graduate School of Comprehensive Human Sciences, University of Tsukuba, Tsukuba, Ibaraki Japan; 5 Department of Medical Research and Management for Musculoskeletal Pain, 22nd Century Medical & Research Center, The University of Tokyo Hospital, Tokyo, Japan; 6 Department of Clinical Medicine, Faculty of Medicine, University of Tsukuba, Tsukuba, Ibaraki Japan; 7 Solutions Center for Health Insurance Claims, Kyoto University Hospital, Kyoto, Japan; 8 Division of Medical Information Technology and Administration Planning, Kyoto University Hospital, Kyoto, Japan; 9 Department of Clinical Epidemiology and Health Economics, School of Public Health, The University of Tokyo, Tokyo, Japan; 10 Department of Health Services Research, Faculty of Medicine, University of Tsukuba, Tsukuba, Ibaraki Japan; 11 Diabetes and Metabolism Information Center, Research Institute, National Center for Global Health and Medicine, Tokyo, Japan

**Keywords:** Medical expenditures, Hip fracture, National Database of Health Insurance Claims and Specific Health Checkups of Japan, Osteoporosis

## Abstract

**Summary:**

Using the nationwide health insurance claims database in Japan, we estimated total annual medical expenditures for fragility hip fracture across the population at 329.2 billion yen (2.99 billion US dollars). Long-term care expenditures were not included. Fragility hip fracture imposes a considerable health economic burden on society in Japan.

**Purpose:**

Fragility hip fracture imposes a substantial health economic burden on society globally. We aimed to estimate medical expenditures for fragility hip fracture using the nationwide health insurance claims database in Japan.

**Methods:**

We included adults aged 60 and over without prior hip fracture who were admitted for fragility hip fracture (i.e., femoral neck or extracapsular) between October 2014 and October 2015 (13 months). Fragility hip fracture was identified through newly assigned disease codes for fracture and procedure codes associated with the fracture. As a proxy for medical expenditures per patient, incremental payments were calculated (i.e., the difference between the total payments 6 months before and after fragility hip fracture). The total payments included health insurance reimbursements and copayments for inpatient and outpatient services. Long-term care expenditures were not included in this study.

**Results:**

We identified 142,361 individuals (28,868 male and 113,493 female) with fragility hip fracture. Mean medical expenditures for fragility hip fracture per patient were 2,550,000 yen (¥) (23,180 US dollars [$]; ¥110 = $1) in male and ¥2,494,000 ($22,670) in female patients, respectively. Total annual medical expenditures for fragility hip fracture across the population were 329.2 billion yen (2.99 billion US dollars): 67.96 billion yen (620 million US dollars) in male and 261.24 billion yen (2.37 billion US dollars) in female patients, respectively.

**Conclusion:**

This is the first study to estimate medical expenditures for hip fracture using the nationwide health insurance claims database, which represents almost all health insurance claims in Japan. Fragility hip fracture inflicts a considerable health economic burden on society in Japan.

**Supplementary Information:**

The online version contains supplementary material available at 10.1007/s11657-022-01096-8.

## Introduction

Fragility hip fracture imposes a substantial health economic burden on society at large. A systematic review and meta-regression analysis, which included 113 studies of over 670,000 patients between 1990 and 2015 [1], has reported a health economic burden inflicted by fragility hip fracture worldwide. The study reported that the pooled mean costs of total health and social care per patient in the 12 months following hip fracture were estimated to be higher than the counterpart estimates for acute coronary syndrome or ischemic stroke. Although the systematic review and meta-regression analysis included studies mainly conducted in North America and Western Europe (44% and 35%, respectively), the authors pointed out that medical expenditures post–hip fracture differ across regions [1].

Three studies in Japan were included in the abovementioned systematic review and meta-regression analysis [2–4]. However, the generalizability of these studies was restricted, as the numbers of hip fracture patients in these three respective studies were only 778 in ten hospitals, 813 in seven hospitals, and 148 in three hospitals. Since 2016, two studies have been conducted on medical expenditures post–hip fracture in Japan using health insurance claims data [5, 6]. In a smaller-scale study published in 2018 using merged health and long-term care insurance claims data from one city (Kashiwa, in Chiba prefecture) to estimate medical expenditures, the number of patients with hip fracture was only 78 [5]. Another study, published in 2018 and using health insurance claims data, included 2415 patients with hip fracture at more than 240 acute care hospitals. Although this was a larger-scale study covering almost one-eighth of the population in Japan, the authors still suggested the possibility of selection bias as one of the limitations of the study (Supplemental Table 1) [6].

To the best of our knowledge, however, no national-level study has been conducted on medical expenditures post–hip fracture in Japan. Using the nationwide health insurance claims database, we aimed to estimate not only mean medical expenditures for fragility hip fracture per patient but also total medical expenditures for fragility hip fracture across the population in Japan.

## Methods

### Data source

In Japan, the universal health insurance system was first implemented in 1961. Since 2008, the copayments have been 30% for adults aged under 70 years, 20% or 30% for those aged between 70 and 74 years, and 10% or 30% for those aged over 75 years [7]. The National Database of Health Insurance Claims and Specific Health Checkups of Japan (NDB) is a national database of health insurance claims and specific health checkup data, and provides all information on universal health insurance, including age, sex, diseases/conditions, diagnostic examinations, medical treatments, surgical procedures, and prescribed medications. The NDB represents almost all health insurance claims in Japan, as Japan has universal health coverage with some exceptions such as accidents covered by automobile liability insurance or workers’ accident compensation outside of the universal health insurance. In addition, local governments provide payments for those on public welfare (less than 2% of the population) [8–16]. We obtained individual-level NDB data for those who met the International Statistical Classification of Diseases and Related Health Problems 10th revision’s (ICD-10’s) criteria for hip fractures (S72, M8435, M8445, M8005, M8085, or M8095) between April 2014 and March 2016. As a personal identification variable, we used ID0 in this analysis [17]. The NDB did not provide the data for the disease codes or procedure codes that had less than 1000 cases per year; however, this did not affect the total payments for each individual. Subject to restrictions, the NDB data may be obtained from the Japanese Ministry of Health, Labour, and Welfare.

### Study population

We included adults aged 60 years and older without prior hip fracture including not only fragility but also open or stress hip fracture (no disease codes for such hip fractures were assigned between April 2014 and September 2014) and who were admitted for new fragility hip fracture between October 2014 and October 2015 (13 months). We chose the period between October 2014 and October 2015 to ensure sufficient pre– and post–hip fracture durations (6 months for each) as described below in the measurements section. We excluded those with less than 6 months of pre–hip fracture duration. In contrast, we included those who died within 6 months post–hip fracture in the base case.

We identified new fragility hip fracture by newly assigned disease codes of hip fracture, including femoral neck and extracapsular (inter- and subtrochanteric), and the surgical procedure codes associated with hip fracture, including total hip replacement, bipolar hip arthroplasty, open or closed reduction and internal fixation, and conservative management (Supplemental Table 2) [5, 13]. We used disease codes instead of ICD-10 codes because disease codes provided more detailed information regarding diagnoses. A study in Japan reported that approximately 98% of patients with hip fracture underwent surgical procedures within 16 days of admission, including the day of admission [18]. Therefore, we excluded those who underwent surgical procedures for hip fracture after 17 days of admission, as it was likely that they were admitted for a different reason and subsequently suffered from hip fracture during hospitalization.

### Measurements

To estimate mean medical expenditures for fragility hip fracture per patient, we used incremental payments as a proxy of medical expenditures; they were calculated as the difference between the total payments 6 months before (i.e., including the month prior to admission for hip fracture and backward) and 6 months after hip fracture (i.e., including the month of admission for hip fracture and forward). We adopted Kilgore et al.’s method, in which the difference between medical expenditures 6 months pre- and post-fracture was calculated on the same individual among the Medicare beneficiaries in the US [19]. In the current study, the total payments consisted of both national health insurance reimbursements and copayments for inpatient and outpatient services. Inpatient services were provided at Diagnosis Procedure Combination/Per-Diem Payment System (DPC/PDPS) and non-DPC/PDPS hospitals. In DPC/PDPS hospitals, two payment systems (i.e., a flat-fee per-diem payment according to diagnostic categories and a fee-for-service payment according to procedures) are incorporated for acute inpatient services. In non-DPC/PDPS hospitals, only a fee-for-service payment system is used [5]. The fee for inpatient prescriptions was included in the inpatient payments. The payments for outpatient services include fees charged by the medical facility and pharmacy. We did not include payments for dental services, meals, living during hospitalization, home nursing care, prosthetic devices, judo therapist, massage, acupuncture, or moxibustion. In Japan, long-term care insurance is separated from health insurance, and long-term care expenditures were not included in this analysis. Outpatient rehabilitation/physiotherapy services are covered either through health insurance or long-term care insurance. We have presented the main payments between April 2014 and March 2016 (fiscal years 2014 and 2015) in Japanese yen (¥). For ease of interpretation, we converted them to US dollars [$] at a rate of ¥110 to $1, which is the approximate mean annual exchange rates between 2014 and 2020 [20].

In addition to the disease codes and surgical procedure codes, we used age (stratified by 5-year ranges as of April 2014), sex, and location of the hospital/clinic (i.e., 47 prefectures, which are official geographical regions in Japan). As a proxy for multimorbidity, we obtained the 2011 updated version of the Charlson Comorbidity Index (CCI) scores by using disease codes within 6 months of admission for hip fracture including the month of admission [21]. We excluded the data in which the diagnoses were provided with a “suspicious” flag, suggesting that tentative diagnoses were placed for the purpose of justifying diagnostic examinations.

### Analysis

In the base case, we calculated mean and median medical expenditures for fragility hip fracture per patient, stratified by sex and including both sexes. Then, we calculated annual medical expenditures for fragility hip fracture across the population by multiplying the number of fractures by mean medical expenditures per patient. As our study population was those who were admitted for new fragility hip fracture over 13 months (i.e., between October 2014 and October 2015), we made an adjustment to obtain annual medical expenditures (i.e., 12 months). In addition, we calculated mean medical expenditures per patient, stratified by age range, anatomical sites of fracture, and surgical procedures. If the disease codes of the hip fractures of multiple anatomical sites were identified, the priority of the classification was the femoral neck followed by extracapsular fractures. If multiple surgical procedures were identified, the priority of the classification was total hip replacement, bipolar hip arthroplasty, open or closed reduction and internal fixation, followed by conservative management. Next, we calculated mean medical expenditures per patient stratified by 47 prefectures in Japan to examine regional differences in Japan. Finally, as a sensitivity analysis, we calculated mean and median medical expenditures, excluding those who died within 6 months after hip fracture including the month of admission for hip fracture. Death was confirmed by the outcomes of inpatient or outpatient services or by the surcharges of the death certificate or end-of-life care. A previous validation study in Japan regarding the accuracy of death information in health insurance claims data showed that claims data can be useful in identifying death, especially inpatient death [22].

## Results

**Fig. 1:**
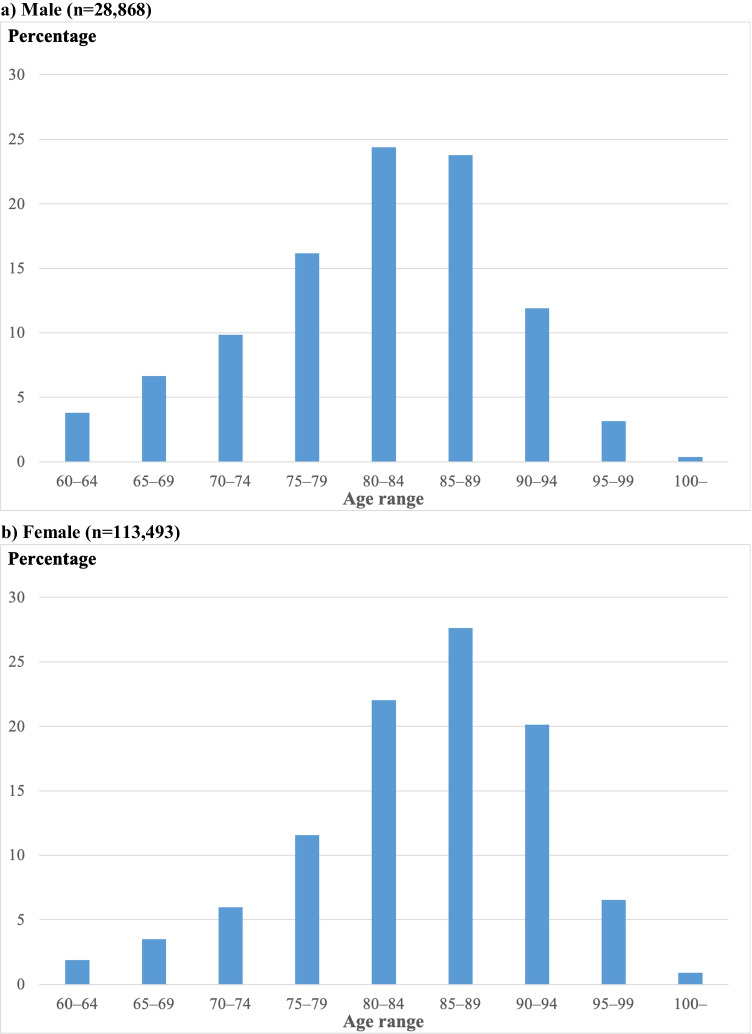
Fragility hip fracture reported between October 2014 and October 2015 (13 months), stratified by age range. **a**) Male (*n* = 28,868), **b**) Female (*n* = 113,493)

Between October 2014 and October 2015, 142,361 individuals (28,868 male and 113,493 female) were admitted for fragility hip fracture. Among those 48.2% male and 49.6% female patients fell within the age range 80–89 (i.e., the sum of 80–84 and 85–89) (Fig. 1). Femoral neck and extracapsular fractures accounted for 58.0% and 42.0%, respectively, in male patients and 56.1% and 43.9%, respectively, in female patients. For femoral neck fracture, bipolar hip arthroplasty was the most common procedure (59.8% in male and 58.9% in female patients), followed by open or closed reduction and internal fixation (33.2% male and 35.1% in female patients). For extracapsular fracture, open or closed reduction and internal fixation were by far the most common procedures (95.2% in male and 95.7% in female patients) (Supplemental Fig. 1). The mean CCI scores were 3.17 in male and 2.56 in female (Supplemental Fig. 2).

Mean and median medical expenditures for fragility hip fracture per patient were ¥2,550,000 ($23,180) and ¥2,369,000 ($21,540) in male and ¥2,494,000 ($22,670) and ¥2,310,000 ($21,000) in female patients, respectively (Table 1; Supplemental Fig. 3). Including both sexes, the mean and median expenditures per patient were ¥2,505,000 ($22,700) and ¥2,321,000 ($21,100), respectively. Total annual medical expenditures for fragility hip fracture across the population were 329.2 billion yen (2.99 billion US dollars): 67.96 billion yen (620 million US dollars) in male and 261.24 billion yen (2.37 billion US dollars) in female patients, respectively.

**Table 1:** Mean medical expenditures for fragility hip fracture per patient: unit 10,000 yen (US dollars [$], 110 yen = $1)

	Male (*n* = 28,868)		Female (*n* = 113,493)	
	Mean	Median	Mean	Median
Total	255.0 ($23,180)	236.9 ($21,540)	249.4 ($22,670)	231.0 ($21,000)
Inpatient	266.8 ($24,260)	246.4 ($22,400)	256.8 ($23,340)	236.7 ($21,520)
Outpatient (medical facility)	− 7.6 (− $690)	− 2.1 (− $190)	− 4.1 (− $380)	− 1.5 (− $130)
Outpatient (pharmacy)	− 4.2 (− $380)	− 1.6 (− $140)	− 3.2 (− $290)	− 1.3 (− $120)

**Fig. 2:**
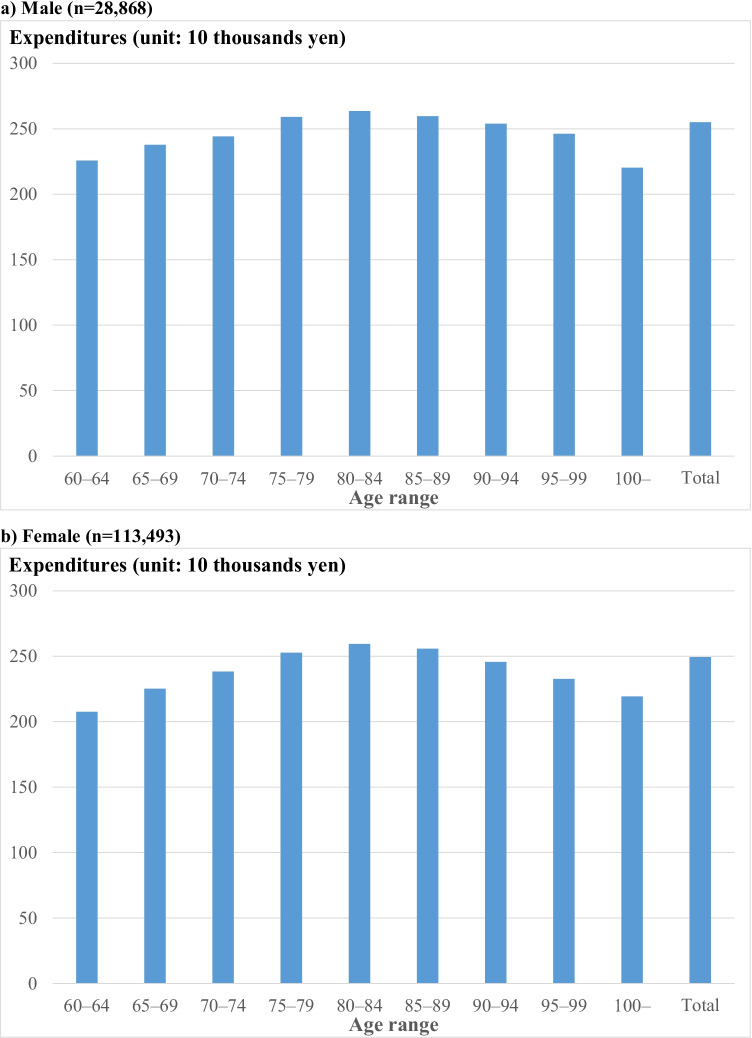
Mean medical expenditures for fragility hip fracture per patient, stratified by age range. **a**) Male (*n* = 28,868), **b**) Female (*n* = 113,493). The figures represent medical expenditures including health insurance reimbursements and copayments for inpatient and outpatient services

Mean medical expenditures per patient were highest in the age range of 80–84 in both sexes (Fig. 2; Supplemental Table 3). For femoral neck fracture, mean medical expenditures per patient were higher in those with bipolar hip arthroplasty than in those with open or closed reduction and internal fixation (¥2,885,000 [$26,230] and ¥2,227,000 [$20,240] in male, ¥2,804,000 [$25,490] and ¥2,187,000 [$19,880] in female patients, respectively). For extracapsular fracture, mean medical expenditures per patient for those with open or closed reduction and internal fixation were ¥2,444,000 ($22,220) in male and ¥2,399,000 ($21,810) in female patients, respectively (Supplemental Table 4). Finally, mean medical expenditures differed across the prefectures, with ¥2,991,000 ($27,190) being the highest and ¥2,088,000 ($18,990) being the lowest in male and ¥3,023,000 ($27,490) being the highest and ¥2,087,000 ($18,980) being the lowest in female patients (Supplemental Fig. 4). In a sensitivity analysis, in which we excluded those who died within 6 months post–hip fracture (11.5% in male and 5.0% in female patients), mean and median medical expenditures per patient were almost the same as those in the primary analysis in both sexes (Supplemental Table 5).

## Discussion

Using the NDB database, which represents almost all health insurance claims in Japan, we estimated medical expenditures for fragility hip fracture in 142,361 individuals (28,868 male and 113,493 female). Total annual medical expenditures for fragility hip fracture across the population were 329.2 billion yen (2.99 billion US dollars): 67.96 billion yen (620 million US dollars) in male and 261.24 billion yen (2.37 billion US dollars) in female patients, respectively.

We used incremental payments as a proxy for mean medical expenditures for fragility hip fracture per patient; they were calculated as the difference between the total payments 6 months before and 6 months after hip fracture. The main advantage of this approach is that the pre-fracture condition of an individual is considered to be a control for the post-fracture condition of the same individual [5, 19]. The incremental differences in the total payments are considered to be dependable estimations of the avoidable costs associated with fracture than the alternatives, such as the summation of all the costs after a fracture or comparison of the costs between the fracture cases and controls [23]. The payments were provided on a monthly basis. Our approach may have resulted in the underestimation of the payments after hip fracture, because we could not take into account the dates of admission for hip fracture. For example, whether an individual was admitted on October 1 or on October 31, the payment in October was considered post–hip fracture in either case. We, however, did not consider this to be a major issue, as 6 months before and after hip fracture are enough durations to capture medical payments post–hip fracture covered by health insurance.

Since 2010, 14 cost-effectiveness analysis articles that included the costs of hip fracture in the Japanese setting have been published (Supplemental Table 6) [24–37]. Among them, nine were published between 2011 and 2019, which cited Kondo et al.’s article published in 2009 as a reference of the cost of hip fracture [4]. Since 2020, five cost-effectiveness analysis articles have been published. Regarding the costs of hip fracture, Taguchi et al.’s article was cited three times and Mori et al.’s article was cited once; both the articles were published in 2018. A cost-effectiveness analysis article written in Japanese cited Ota’s article (also written in Japanese) published in 2002 [5, 6, 38]. Ishizaki et al., Hirose et al., and Kondo et al.’s studies, published before 2009, summed up all the costs after fracture, while Taguchi et al. and Mori et al.’s studies calculated the incremental differences in the total payments before and after hip fracture [2–6]. (Supplemental Table 1).

Our results were very similar to those of a previous study by Mori et al. (coauthored by a few authors of the current study) using health insurance claims data in the city of Kashiwa, the population of which was approximately 400,000 in 2012, located in Chiba prefecture. In this study, the target population was adults aged 75 years and over admitted for hip fracture to non-DPC/PDPS hospitals between April 2012 and September 2013. Mean medical expenditures per patient were calculated using almost the same method as that used in the current study (i.e., the difference between the total payments 6 months before and after hip fracture of the same individuals). The study included only 78 individuals and showed that estimated mean medical expenditures per patient were ¥2,600,000 or $29,500 (¥88 = $1) [5]. With the availability of the NBD database that represents almost all health insurance claims in Japan, the present study confirmed the generalizability of the results of the previous study in a single city. In addition, we estimated total annual medical expenditures for fragility hip fracture across the population in Japan.

Mean medical expenditures for fragility hip fracture per patient were the highest in the age range of 80–84 in both sexes in our study. These results were not consistent with total annual mean medical expenditures for any diseases or conditions per patient in Japan in the fiscal year 2015, in which total annual mean medical expenditures (i.e., the sum of inpatient and outpatient medical services and pharmacy) per patient for those aged 85 years and over were higher (¥1,100,000 or $10,000  in male and ¥954,000 or $8670 in female) than for those aged between 80 and 84 years (¥965,000 or $8770 in male and ¥810,000 or $7360 in female) [39]. These results may be owing to the fact that invasive procedures and treatments may be withheld for those with hip fracture aged 85 and over, and especially for those aged beyond 90. A previous study in ten teaching hospitals in Japan between 1996 and 2000 showed that mean medical expenditures post–hip fracture per patient was $15,012 or ¥1,801,000 ($1 = ¥120) in individuals aged 65–74 years (*n* = 190), $14,744 or ¥1,769,000 in individuals aged 75–84 years (*n* = 356), and $13,690 or ¥1,643,000 in individuals aged 85 years and over (*n* = 232) (*p*-value of Kruskal–Wallis test: 0.006). These findings were consistent with ours that mean medical expenditures for those aged 85 years and over were not higher than expenditures for those aged between 75 and 84 years, but not consistent with our finding that expenditures for those aged between 65 and 74 years were higher than expenditures for those aged between 75 and 84 years [2]. However, it is difficult to interpret the results, comparing our study with the study that used the data between 1996 and 2000.

For femoral neck fracture, bipolar hip arthroplasty was the most common procedure, followed by open or closed reduction and internal fixation. Mean medical expenditures for hip fracture per patient were higher in those with bipolar hip arthroplasty (¥2,885,000 [$26,230] in male and ¥2,804,000 [$25,490] in female patients) than in those with open or closed reduction and internal fixation (¥2,227,000 [$20,240] in male and ¥2,187,000 [$19,880] in female patients) (Supplemental Table 4). The payments for the procedures, including both reimbursements and copayments, were ¥195,000 ($1,770) for bipolar hip arthroplasty and ¥188,000 ($1,710) for open or closed reduction and internal fixation during the study period (i.e., the fiscal year 2014–2015); the difference in the payments (¥7000, or $70) was not enough to explain the difference between the expenditures [40]. For extracapsular fracture, open or closed reduction and internal fixation were by far the most common procedures, and mean medical expenditures per patient for those with open or closed reduction and internal fixation were ¥2,444,000 ($22,220) in male and ¥2,399,000 ($21,810) in female patients. Mean medical expenditures per patient for open or closed reduction and internal fixation were higher for those with extracapsular fracture than for those with femoral neck fracture.

Mean medical expenditures for hip fracture per patient in each prefecture have been presented. Overall, there seem to be a similar trend in mean medical expenditures for fragility hip fracture per patient stratified by prefecture to the regional differences in total annual medical expenditures for any diseases or conditions per patient in Japan (i.e., higher expenditures in the western part of Japan than in the eastern part) [41].

Since 2019, several studies have reported osteoporotic fracture using the NDB database. One study examined the association between seasonality and incident fracture at different sites [8]. Using a case-crossover study design, incident fragility fracture, the associations of statin use with fragility fracture, and the associations of central nervous system agents with fragility fracture have been reported [9–12]. The studies examining the testing rates of bone mineral density and pharmacotherapy after hip or vertebral fracture and persistence to pharmacotherapy for osteoporosis have been presented [14, 15]. Aggregated NDB data have been used to examine the annual incidence rates of hip fracture [13, 16]. To the best of our knowledge, our study is the first health economic study regarding osteoporotic fracture using the NDB database in Japan, and it has revealed the status of medical expenditures for hip fracture in Japan.

This study has several limitations. First, health insurance claims data were not intended to be developed for research purposes; therefore, they are subject to coding errors and misclassifications. We have made great efforts to identify new fragility hip fracture by combining newly assigned disease codes of hip fracture and surgical procedures associated with hip fracture. Second, it is important to note that we only included direct costs post-fracture and that there are also considerable additional costs associated with social aspects of care. In Japan, long-term care insurance is separated from health insurance and covers social aspects of care to some extent. Using the NDB database, we calculated the differences between the total payments 6 months before and after hip fracture to estimate medical expenditures for hip fracture. Although long-term care plays a major role beyond 6 months post–hip fracture, we did not include long-term care expenditures in this study, as the NDB database provides information regarding health insurance but does not contain information regarding long-term care insurance [5]. Future studies are warranted when comprehensive data including both medical and long-term care expenditures post–hip fracture are available for academic use. Third, although we estimated total annual medical expenditures for fragility hip fracture across the population in Japan, it would be difficult to compare these expenditures with the annual expenditures for other diseases because of the lack of such counterpart studies (to the best of our knowledge) in Japan. Further studies will be warranted to reveal such expenditures using the NDB database. Finally, although we found several differences in medical expenditure post–hip fracture among groups, examining the factors associated with these differences was beyond the scope of this study.

Despite these limitations, our study had several strengths. This is the first study to estimate medical expenditures for hip fracture using the nationwide health insurance claims database, which represents almost all health insurance claims in Japan and estimated not only mean medical expenditures for fragility hip fracture per patient but also annual medical expenditures for fragility hip fracture across the population. In addition, we calculated mean medical expenditures for fragility hip fracture per patient stratified by sex and further by age range, anatomical sites and surgical procedures, and prefecture. Our results, especially sex- and age-range-stratified expenditures, may prove to be useful for future cost-effectiveness analyses in Japan, where the annual incidence rate of hip fracture has increased [42]. Cost-effectiveness analysis has become even more important in Japan, as in 2019, the Japanese Ministry of Health, Labour, and Welfare officially introduced health technology assessment by implementing cost-effectiveness analysis to determine price adjustments for drugs and medical devices [43].

In conclusion, using the NDB database representative of almost all health insurance claims in Japan, we estimated total annual medical expenditures for fragility hip fracture across the population at 329.2 billion yen (2.99 billion US dollars). Fragility hip fracture imposes a considerable health economic burden on society in Japan.

## Supplementary Information

Below is the link to the electronic supplementary material.

Supplementary file1 (DOCX 554 KB)

## Data Availability

The data and material may be obtained from the Japanese Ministry of Health, Labour, and Welfare.

## Code availability

Considered upon request.
