# Supplementary material for: Medical expenditures for fragility hip fracture in Japan: a study using the nationwide health insurance claims database
Source: Arch Osteoporos. 2022 Apr 11;17(1):61. doi: 10.1007/s11657-022-01096-8 (PMC9001568; doi:10.1007/s11657-022-01096-8)

**Supplemental Table 1: Previous studies estimating medical expenditures for hip fracture in Japan**

|  | **First author (year published)** | **Study period, population (n)** | **Mean expenditures per patient* (unit: 10 thousands yen, 110 yen=$1)**** | **Method** |
| --- | --- | --- | --- | --- |
| (1) | Ishizaki et al. [1] | Jan 1996 to Aug 2000,  four privately owned leading teaching hospitals (n=778) | 173.9 ($19,130) | Total charges were calculated by summing any charge billed during the hospital stay. |
| (2) | Hirose et al. [2] | Sept 2003 to Dec 2005, seven member  hospitals of the Japanese National Hospital Organization (n=813) | $15,800 (only the amount in US dollars is presented, and currency exchange rate is not available) | Hospitalization costs included all charges for the operation, laboratory tests, diagnostic imaging, ward costs, and expenditures incurred for treatment of postoperative complications. |
| (3) | Kondo et al. [3] | Aug 2005 to Sept 2007, three hospitals including a university hospital and two private general community hospitals (bed size ranged from 200 to 700) (n=113) | 220.8 ($24,290) | Approximate cost was calculated by the number of hospitalization days and average insurance payment per day. |
| (4) | Mori et al. [4] | Apr 2012 to Sept 2013, healthcare insurance claims data for adults aged ≥ 75 years in the City of Kashiwa (n=78) | 260.0 ($28,600) | Medical expenditures were calculated by the difference between total payments six months before and after hip fracture in the same individuals. |
| (5) | Taguchi et al. [5] | Apr 2008 to Dec 2016, the medical claim database provided by Medical Data Vision Co. Ltd. (MDV; Tokyo, Japan) in more than 240 acute-care hospitals (n=12,898; 2143 male, 10755 female) | 160.3 ($17,630) in male, 172.6 ($18,990) in female patients | The total medical cost was estimated by subtracting the treatment costs within one year before index fracture from the treatment costs within one year after index fracture. Costs were calculated by multiplying the amount used by the unit price for each resource use record from public health care payer’s perspective. |

* Costs were not adjusted to yen in a specific year. The consumer index price during the study periods ranged from 97% to 103% compared with the price in the fiscal year 2015. (reference: Statistics Bureau, Ministry of Internal Affairs and Communications. Consumer Price Index. https://www.stat.go.jp/data/cpi/2015/).

** Unless otherwise specified.

(1) Ishizaki T, Imanaka Y, Oh E, Kuwabara K, Hirose M, Hayashida K, Harada Y (2004) Association of hospital resource use with comorbidity status and patient age among hip fracture patients in Japan. Health Policy 69(2):179–187. <https://doi.org/10.1016/j.healthpol.2003.12.018>

(2) Hirose J, Mizuta H, Ide J, Nakamura E, Takada K (2008) E-PASS for predicting postoperative risk with hip fracture: a multicenter study. Clin Orthop Relat Res 466(11):2833–2841. <https://doi.org/10.1007/s11999-008-0377-2>

(3) Kondo A, Zierler BK, Isokawa Y, Hagino H, Ito Y (2009) Comparison of outcomes and costs after hip fracture surgery in three hospitals that have different care systems in Japan. Health Policy 91(2):204–210. <https://doi.org/10.1016/j.healthpol.2008.12.006>

(4) Mori T, Tamiya N, Jin X, Jeon B, Yoshie S, Iijima K, Ishizaki T (2018) Estimated expenditures for hip fractures using merged healthcare insurance data for individuals aged≥ 75 years and long-term care insurance claims data in Japan. Arch Osteoporos 13(1):37. <https://doi.org/10.1007/s11657-018-0448-2>

(5) Taguchi Y, Inoue Y, Kido T, Arai N (2018) Treatment costs and cost drivers among osteoporotic fracture patients in Japan: a retrospective database analysis. Arch Osteoporos 13(1):45. <https://doi.org/10.1007/s11657-018-0456-2>

**Supplemental Table 2: Disease and surgical procedure codes to identify hip fractures**

| **Disease codes** | **Names of fractures** |
| --- | --- |
| **Femoral neck** | |
| 8208009  8837297 8837303 8837305 8837311 | Femoral neck  Transcervical femoral neck  Medial femoral neck  Subcapital femur  Lateral femoral neck |
| **Extracapsular (intertrochanteric and subtrochanteric)** | |
| 8837314  8837298  8837299  8837300  8837943  8837944  8837945 | Trochanteric  Pertrochanteric  Intertrochanteric  Basicervical  Subtrochanteric  Intertrochanteric  Pertrochanteric |
|  | |
| **Procedure codes** | **Names of surgical procedures** |
| 150050410 | Total hip replacement |
| 150049510 | Bipolar hip arthroplasty |
| 150019210 | Open surgery for femur fracture |
|  | Conservative management |
| 150016710  150243010  140029210  140048010 | Closed reduction  Direct traction with wire (day 1)  Direct traction with wire (day 2 and beyond)  Indirect traction |

**Supplemental Table 3: Mean and median medical expenditures for fragility hip fracture per patient, stratified by age range and sex**

**(unit: 10 thousands yen, 110 yen=1 US dollars [$])**

|  | **Male (n=28,868)** | | **Female (n=113,493)** | |
| --- | --- | --- | --- | --- |
| **Age range** | **Mean** | **Median** | **Mean** | **Median** |
| 60–64 | 225.7 ($20,520) | 197.9 ($17,990) | 207.5 ($18,870) | 190.5 ($17,320) |
| 65–69 | 237.8 ($21,620) | 210.0 ($19,090) | 225.3 ($20,480) | 203.8 ($18,530) |
| 70–74 | 244.2 ($22,200) | 221.8 ($20,160) | 238.3 ($21,660) | 220.2 ($20,020) |
| 75–79 | 259.1 ($23,550) | 239.8 ($21,800) | 252.8 ($22,980) | 236.1 ($21,470) |
| 80–84 | 263.6 ($23,960) | 248.9 ($22,630) | 259.4 ($23,580) | 243.7 ($22,160) |
| 85–89 | 259.7 ($23,610) | 245.3 ($22,300) | 255.8 ($23,260) | 239.0 ($21,730) |
| 90–94 | 253.9 ($23,080) | 239.8 ($21,800) | 245.7 ($22,340) | 227.0 ($20,640) |
| 95–99 | 246.2 ($22,380) | 232.3 ($21,120) | 232.6 ($21,150) | 209.4 ($19,040) |
| 100– | 220.2 ($20,020) | 199.7 ($18,150) | 219.3 ($19,940) | 198.9 ($18,080) |
| Total | 255.0 ($24,090) | 236.9 ($21,800) | 249.3 ($23,360) | 231.0 ($21,340) |

**Supplemental Table 4: Medical expenditures for fragility hip fracture per patient, stratified by anatomical sites and surgical procedures,**

**(unit: 10 thousands yen, 110 yen= 1 US dollars [$]))**

|  | **Male (n=28,868)** | | **Female (n=113,493)** | |
| --- | --- | --- | --- | --- |
|  | **Mean** | **Median** | **Mean** | **Median** |
| **Femoral neck** | n=16,752 (58.0%) | | n=63,677 (56.1%) | |
| Total hip replacement | 317.4 ($28,860) | 277.8 ($25,260) | 308.5 ($28,050) | 272.6 ($24,780) |
| Bipolar hip arthroplasty | 288.5 ($26,230) | 266.7 ($24,250) | 280.4 ($25,490) | 258.7 ($23,520) |
| Open or closed reduction and internal fixation | 222.7 ($20,240) | 199.0 ($18,090) | 218.7 ($19,880) | 193.2 ($17,560) |
| Conservative management | 215.9 ($19,630) | 203.2 ($18,470) | 224.1 ($20,370) | 214.1 ($19,460) |
| Total | 257.8 ($23,440) | 237.8 ($21,620) | 256.5 ($23,320) | 236.6 ($21,510) |
|  | | | | |
| **Extracapsular (inter and subtrochanteric)** | n=12,116 (42.0%) | | n=49,816 (43.9%) | |
| Total hip replacement or bipolar hip arthroplasty | 375.5 ($34,140) | 359.7 ($32,700) | 365.8 ($33,250) | 358.3 ($32,580) |
| Open or closed reduction and internal fixation | 244.4 ($22,220) | 226.9 ($20,630) | 239.9 ($21,810) | 222.2 ($20,200) |
| Conservative management | 207.8 ($18,890) | 199.1 ($18,100) | 214.8 ($19,520) | 208.6 ($18,970) |
| Total | 241.0 ($21,910) | 223.7 ($20,330) | 240.3 ($21,840) | 229.7 ($20,270) |

**Supplemental Table 5: Sensitivity analysis, medical expenditures for fragility hip fracture per patient, excluding those who died within six months post hip fracture (unit: 10 thousands yen, 110 yen=1 US dollars [$]))**

|  | **Male** | | **Female** | |
| --- | --- | --- | --- | --- |
| **Numbers (% of the base case)** | 25,562 (88.5%) | | 107,769 (95.0%) | |
|  | **Mean** | **Median** | **Mean** | **Median** |
| **Medical expenditures** | 261.1 ($23,740) | 241.2 ($21,930) | 251.7 ($22,880) | 232.9 ($21,180) |

**Supplemental Table 6: Cost-effectiveness analyses including the cost of hip fracture in Japan since 2010**

| Year published | Cost-effectiveness analysis article | **Reference article for cost of hip fracture * (Year)** |
| --- | --- | --- |
| 2011 | Goto S, Komaba H, Moriwaki K, et al. Clinical efficacy and cost-effectiveness of lanthanum carbonate as second-line therapy in hemodialysis patients in Japan. Clinical Journal of the American Society of Nephrology 2011; 6(6): 1375–84. | Kondo et al. [3] |
| 2012 | Komaba H, Moriwaki K, Goto S, et al. Cost-effectiveness of cinacalcet hydrochloride for hemodialysis patients with severe secondary hyperparathyroidism in Japan. American journal of kidney diseases 2012; 60(2): 262–71. | Kondo et al. [3] |
| 2013 | Moriwaki K, Komaba H, Noto S, et al. Cost‐effectiveness of alendronate for the treatment of osteopenic postmenopausal women in Japan. Journal of Bone and Mineral Research 2013; 28(2): 395–403. | Kondo et al. [3] |
| 2017 | Moriwaki K, Noto S. Economic evaluation of osteoporosis liaison service for secondary fracture prevention in postmenopausal osteoporosis patients with previous hip fracture in Japan. Osteoporosis International 2017; 28(2): 621–32. | Kondo et al. [3] |
| 2017 | Yoshimura M, Moriwaki K, Noto S, Takiguchi T. A model-based cost-effectiveness analysis of osteoporosis screening and treatment strategy for postmenopausal Japanese women. Osteoporosis International 2017; 28(2): 643–52. | Kondo et al. [3] |
| 2017 | Mori T, Crandall C, Ganz D. Cost-effectiveness of denosumab versus oral alendronate for elderly osteoporotic women in Japan. Osteoporosis International 2017; 28(5): 1733–44. | Kondo et al. [3] |
| 2017 | Moriwaki K, Mouri M, Hagino H. Cost-effectiveness analysis of once-yearly injection of zoledronic acid for the treatment of osteoporosis in Japan. Osteoporosis International 2017; 28(6): 1939–50. | Kondo et al. [3] |
| 2018 | Yoshizawa T, Nishino T, Okubo I, Yamazaki M. Cost-effectiveness analysis of drugs for osteoporosis treatment in elderly Japanese women at high risk of fragility fractures: comparison of denosumab and weekly alendronate. Archives of osteoporosis 2018; 13(1): 1–11. | Kondo et al. [3] |
| 2019 | Moriwaki K, Fukuda H. Cost-effectiveness of implementing guidelines for the treatment of glucocorticoid-induced osteoporosis in Japan. Osteoporosis International 2019; 30(2): 299–310. | Kondo et al. [3] |
| 2020 | Kato G, Kurachi Y. Cost-effective Analysis of Exercise Programs Designed for Fall. Prevention among Healthy Younger Old Community-dwelling Adults (Japanese). Journal of the Japanese Physical Therapy Association 2020; 47(5): 420–430. | Ota et al. [6] |
| 2021 | Hagino H, Tanaka K, Silverman S, et al. Cost effectiveness of romosozumab versus teriparatide for severe postmenopausal osteoporosis in Japan. Osteoporosis International 2021; 32(10): 2011–2021. | Mori et al. [4] |
| 2021 | Mori T, Crandall CJ, Fujii T, Ganz DA. Cost-effectiveness of sequential daily teriparatide/weekly alendronate compared with alendronate monotherapy for older osteoporotic women with prior vertebral fracture in Japan. Archives of Osteoporosis 2021; 16(1): 72. | Taguchi et al. [5] |
| 2021 | Mori T, Crandall CJ, Fujii T, Ganz DA. Cost-effectiveness of zoledronic acid compared with sequential denosumab/alendronate for older osteoporotic women in Japan. Archives of osteoporosis 2021; 16(1): 113. | Taguchi et al. [5] |
| 2021 | Takura T, Yuasa A, Yonemoto N, et al. Cost-Effectiveness Analysis of the Treatment Strategies with or without Opioid Medications in Surgery-Eligible Patients with Osteoarthritis in Japan. PharmacoEconomics-Open 2021; 6: 33–45. | Taguchi et al. [5] |

* Cost-effectiveness analysis articles cited one of the following articles as a reference of the cost of hip fracture

(3) Kondo A, Zierler BK, Isokawa Y, Hagino H, Ito Y (2009) Comparison of outcomes and costs after hip fracture surgery in three hospitals that have different care systems in Japan. Health Policy 91(2):204–210. <https://doi.org/10.1016/j.healthpol.2008.12.006>

(4) Mori T, Tamiya N, Jin X, Jeon B, Yoshie S, Iijima K, Ishizaki T (2018) Estimated expenditures for hip fractures using merged healthcare insurance data for individuals aged≥ 75 years and long-term care insurance claims data in Japan. Arch Osteoporos 13(1):37. <https://doi.org/10.1007/s11657-018-0448-2>

(5) Taguchi Y, Inoue Y, Kido T, Arai N (2018) Treatment costs and cost drivers among osteoporotic fracture patients in Japan: a retrospective database analysis. Arch Osteoporos 13(1):45. <https://doi.org/10.1007/s11657-018-0456-2>

(6) Ota T, Harada A, Tokuda H (2002) Cost-effectiveness of hip fracture in Japan (Japanese). Nihon Ronen Igakkai Zasshi 39(5):483–488. <https://doi.org/10.3143/geriatrics.39.483>

**Supplemental Figure 1-1: Surgical procedures for femoral neck fracture**

**a) Male (n=16,752)**

**b) Female (n=63,677)**

**Supplemental Figure 1-2: Surgical procedures for extracapsular (inter and subtrochanteric) fractures**

**a) Male (n=12,116)**

**b) Female (n=49,816)**

**Supplemental Figure 2: Mean Charlson Comorbidity Index scores before fragility hip fracture**

**a) Male (n=28,868)**

**b) Female (n=113,493)**

**Supplemental Figure 3: Difference in the total payments between six months before and after fragility hip fracture per patient**

**a) Male (n=28,868)**

**b) Female (n=113,493)**

Figure note: the total payments consisted of both national health insurance reimbursements and copayments for inpatient and outpatient services.

**Supplemental Figure 4: Mean medical expenditures for fragility hip fracture per patient, stratified by prefectures**

**a) Male (n=28,868)**


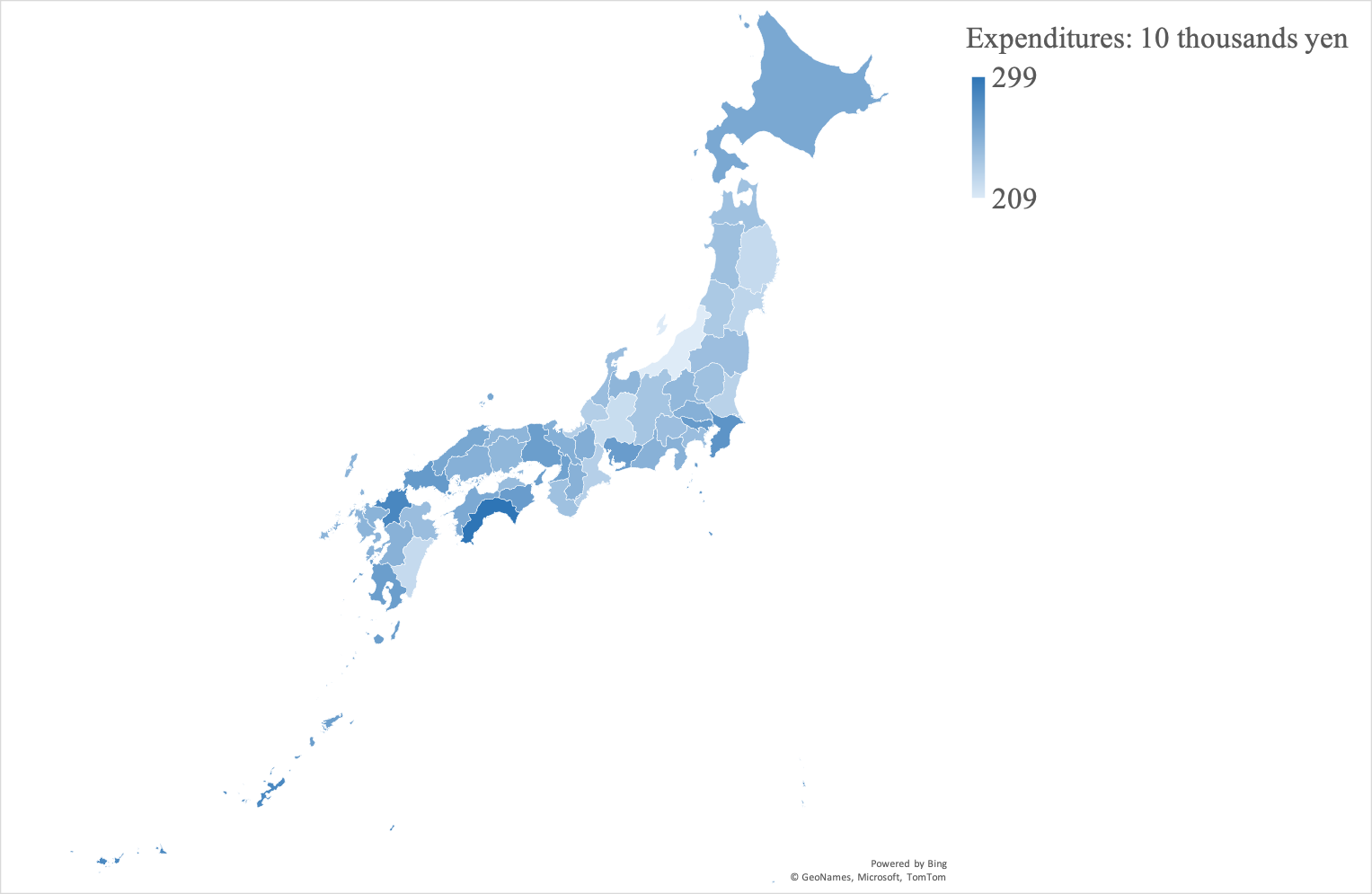


**b) Female (n=113,493)**


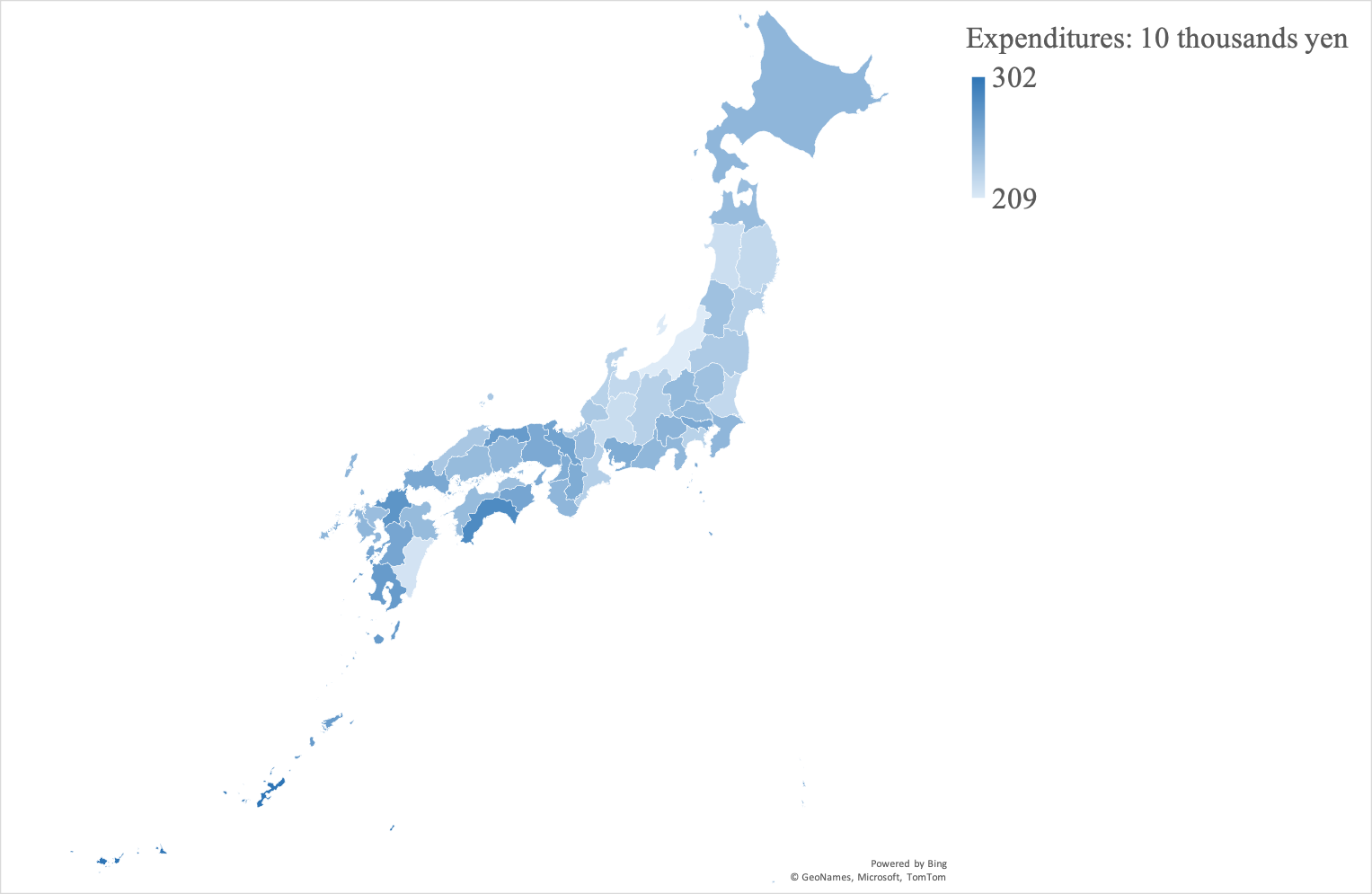

Supplement: Supplementary file 1 — Supplementary file1 (DOCX 554 KB) [file 11657_2022_1096_MOESM1_ESM.docx]
